# Impact of *BRCA* Alterations on Androgen Receptor Pathway Inhibition Treatment Outcomes in Advanced Prostate Cancer

**DOI:** 10.1158/2767-9764.CRC-26-0477

**Published:** 2026-10-12

**Authors:** Ruchi Chaudhary, Karen A. Urtishak, Michael Gormley, Angela Lopez-Gitlitz, Suneel Mundle, Ademi Santiago-Walker, Sharon McCarthy, Fei Shen, Geoffrey T. Gotto

**Affiliations:** 1 Johnson & Johnson Innovative Medicine, Spring House, Pennsylvania.; 2 Johnson & Johnson, Los Angeles, California.; 3 Johnson & Johnson, Raritan, New Jersey.; 4 Southern Alberta Institute of Urology, University of Calgary, Calgary, Canada.; 5 The Vancouver Prostate Centre, University of British Columbia, Vancouver, Canada.

## Abstract

Androgen receptor pathway inhibitors (ARPI) are frequently used to treat advanced prostate cancer. Understanding the impact of homologous recombination repair (HRR) alterations on ARPI treatment outcomes is critical for optimizing treatment. We conducted a retrospective analysis of pooled data from five randomized trials of ARPIs (apalutamide or abiraterone acetate) across prostate cancer disease settings. Patients received ARPI-based regimens or androgen deprivation therapy (ADT) alone. HRR alterations were determined using targeted panel or whole-exome sequencing of diagnostic tissue or plasma samples. Radiographic progression-free survival (rPFS) and overall survival (OS) were compared between biomarker groups. Of 608 patients treated with ARPI-based regimens, *BRCA*-altered patients were at higher risk of progression (HR = 1.8; 95% confidence interval, 1.16–2.79; *P* value = 0.009) and mortality [HR = 1.6 (1.02–2.34); *P* value = 0.038] than those with *BRCA* wild type. HRR-altered patients had shorter rPFS than HRR wild-type patients [HR = 1.7 (1.2–2.3); *P* value = 0.001]. *BRCA*-altered patients had shorter OS compared with HRR wild-type patients [HR = 1.6 (1.03–2.36); *P* value = 0.036]. The same results were observed in the full cohort of 878 patients treated with next-generation ARPI or ADT alone. In 115 HRR-altered patients, addition of ARPIs to ADT significantly prolonged rPFS [HR = 0.42 (0.2–0.89); *P* value = 0.023] and OS [HR = 0.45 (0.23–0.91); *P* value = 0.025] versus ADT alone. Although patients with HRR gene alterations benefit from the addition of ARPIs to ADT, they generally have worse outcomes with androgen receptor–directed therapy than HRR wild-type patients, with patients harboring *BRCA* alterations doing particularly poorly. These findings underline the unmet need for new and combination approaches for these patients.

**Significance:**

ARPIs are standard-of-care treatment for advanced prostate cancer, but prostate cancer is a molecularly and clinically heterogeneous disease. HRR gene alterations are linked to more aggressive disease. We retrospectively analyzed pooled data from five randomized trials of next-generation ARPIs to assess the impact of HRR alterations on outcomes. Our study highlights an unmet need for improved therapies in patients with HRR-altered prostate cancer treated with ARPIs.

## Introduction

Androgen receptor (AR) is the main oncogenic driver of prostate cancer, and primary standard-of-care (SOC) treatments for advanced prostate cancer target AR (1). However, several studies have described the heterogeneity of prostate cancer at the molecular level and in clinical outcomes (2–6). Defects in homologous recombination repair (HRR) genes, particularly *BRCA1/2*, are of relevance as they confer sensitivity to PARP inhibitors (PARPi; refs. 7, 8). The success of PARPis in ovarian and breast cancers paved the way for studies of HRR-defective tumors of prostate origin. Several recent approvals in the metastatic castration-resistant or castration-sensitive prostate cancer setting are based on improved outcomes of adding PARPis to next-generation AR pathway inhibitors (ARPI) and androgen deprivation therapy (ADT; refs. 9–17). An important unanswered question is whether patients with HRR-deficient tumors receive equal benefit from targeting AR compared with those with non-HRR–deficient tumors. We addressed this question by pooling patients from five next-generation ARPI [apalutamide (Apa) and abiraterone acetate (Abi)] trials to enable a statistically powered meta-analysis study (18–22).

HRR deficiency is mainly assessed by detecting mutations in HRR pathway genes, particularly *BRCA1* or *BRCA2*, using targeted sequencing panels. Alternative approaches include evaluating genome-wide structural aberrations—such as loss of heterozygosity, telomeric allelic imbalance, and large-scale transitions—or applying gene expression–based homologous recombination deficiency (HRD) signatures (23–26). In alignment with current clinical practice guidelines, our study focused on detecting HRR pathway mutations to assess HRR deficiency, as this method remains the most validated and widely adopted for patient stratification and therapeutic decision-making.

The main objective of this retrospective study was to evaluate the impact of alterations in HRR genes on response to SOC treatment with next-generation ARPI–based therapies in diagnoses spanning metastatic castration-sensitive prostate cancer (mCSPC), nonmetastatic castration-resistant prostate cancer (nmCRPC), or metastatic castration-resistant prostate cancer (mCRPC). Of note, patients received background ADT along with next-generation ARPI as appropriate. To explore the impact of *BRCA* (*BRCA1 or BRCA2*) versus other genes in the HRR gene family, biomarker groups were defined as (i) *BRCA* altered (*BRCA-alt*) versus *BRCA* wild type (*BRCA-wt*), (ii) HRR altered (*HRR-alt*) versus HRR wild type (HRR-wt), and (iii) *BRCA* altered (*BRCA-alt*) versus *BRCA* wild type but other HRR gene altered (*BRCA-wt/HRR-alt*) versus HRR wild type (*HRR-wt*). Although the focus of this meta-analysis was to combine patients with different diagnoses to increase statistical power, we also explored the effects of BRCA- or HRR-altered versus wild-type status in individual disease settings. In addition, the impact of individual HRR gene alterations on outcomes was also explored. The effect of *BRCA* or HRR alteration status on clinical outcomes was evaluated in patients treated with regimens containing next-generation ARPI as well as in the complete study population of patients treated with next-generation ARPI–based regimes or ADT alone. Lastly, we explored whether adding regimens including treatment intensification of next-generation ARPI to ADT offered benefit to HRR-treated patients by comparing the outcomes of treatment groups in HRR- or *BRCA*-altered patients.

## Materials and Methods

### Study design and patient selection

We conducted a retrospective analysis of pooled data from five randomized clinical trials of next-generation ARPI–based regimens that enrolled patients between February 2013 and September 2020. All patients received treatment with an Apa- or Abi-based regimen (with background ADT) or ADT alone for mCSPC, nmCRPC, or mCRPC. The five trials included SPARTAN (NCT01946204), TITAN (NCT02489318), LATITUDE (NCT01715285), ACIS (NCT02257736), and PCR2023 (NCT01867710; Fig. 1). SPARTAN, TITAN, LATITUDE, and ACIS were phase III trials, whereas PCR2023 was phase II. Patient inclusion criteria and clinical endpoints have been reported in prior publications (18–22).

**Figure 1.**
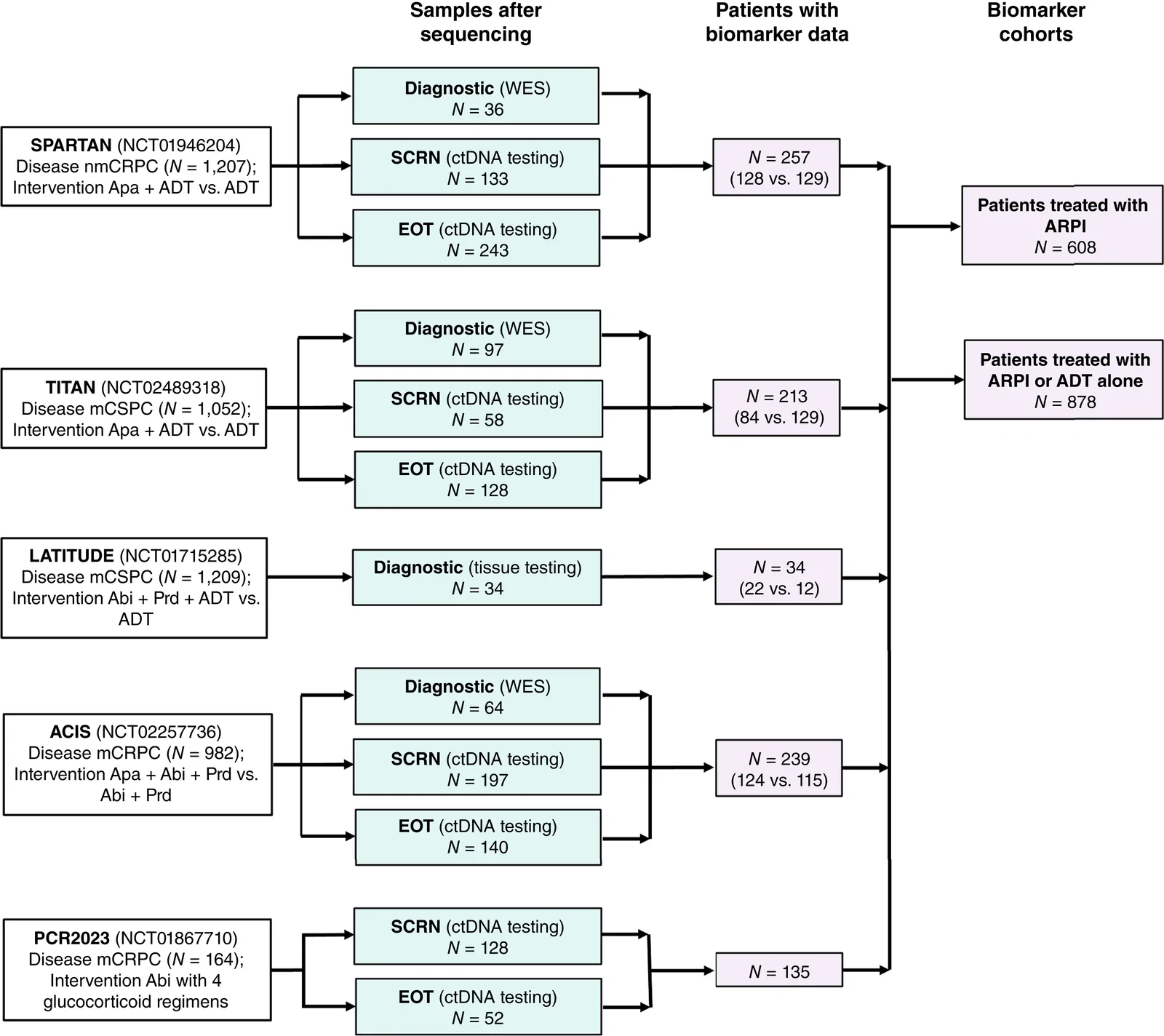
Study design. Five ARPI trials from advanced prostate cancer. NGS data integration from five trials. Samples are diagnostic tissues or on-trial SCRN or EOT plasma. All samples or timepoints are not available for all patients. Prd, prednisone.

A total of 878 patients with diagnostic biopsy tissue or blood (plasma) collected at trial screening (SCRN) or end of treatment (EOT) were included in the analysis. Targeted panel sequencing or whole-exome sequencing (WES) was performed to detect gene alterations. WES samples were required to have matched whole blood for somatic variant calling through tumor–normal analysis. For SPARTAN, TITAN, and ACIS, diagnostic tissue and SCRN and EOT plasma samples were included in the analysis. For LATITUDE, only diagnostic tissue samples and for PCR2023, plasma samples were available (Fig. 1). Overlapping longitudinal samples were rarely collected from the same patients. Across SPARTAN, TITAN, and ACIS, only 71 of 709 (10%) patients had both diagnostic tissue and an SCRN or EOT plasma sample available for testing. *HDAC2* was not included in the next-generation sequencing (NGS) panel for LATITUDE, in which four of 34 patients had HRR alterations in the remaining HRR genes. Consequently, HRR status for all eight genes could only be determined in 848 of 878 patients. For frequency calculations, only SPARTAN, TITAN, ACIS, and PCR2023 (*N* = 844) were included. Cohorts were selected appropriately for the analyses.

A meta-analysis was performed on data from multiple sequencing efforts conducted over the past decade. For several of these efforts, the exact counts of samples submitted for sequencing are unavailable. The analysis encompassed various sample types (e.g., blood and tissue) and NGS pipelines (e.g., WES and targeted panels), each with distinct failure rates. Notably, a WES pair is considered failed if either the formalin-fixed, paraffin-embedded (FFPE) tumor sample or the matched blood sample does not meet quality criteria. Within PCR2023 sample testing, the failure rate for SCRN ctDNA testing was 15% (151 SCRN blood samples submitted, with 128 being successfully processed), whereas the failure rate for EOT ctDNA testing was 8.8% (57 EOT blood samples submitted, with 52 being successfully processed). In the TITAN trial, 32.6% of FFPE and matched blood pairs failed sequencing (144 pairs submitted, with 97 being successfully processed).

Baseline demographic and clinical characteristics of the patients included in the meta-analysis are described in Table 1. The review board at each participating institution approved the trial, which was conducted in accordance with the current International Conference on Harmonization guidelines for Good Clinical Practice and the principles of the Declaration of Helsinki. All the patients provided written informed consent.

**Table 1.** Baseline demographic and clinical characteristics by biomarker group.

Characteristic	All	*BRCA*-alt	*BRCA*-wt	BRCA-wt/HRR-alt	HRR-alt	HRR-wt
	*n* = 878	*n* = 50	*n* = 828	*n* = 69	*n* = 119	*n* = 729
Age at study entry	​	​	​	​	​	​
<65	217 (24.7%)	12 (24%)	205 (24.8%)	17 (24.6%)	29 (24.4%)	172 (23.6%)
65–74	367 (41.8%)	22 (44%)	345 (41.7%)	29 (42%)	51 (42.9%)	301 (42.1%)
≥75	294 (33.5%)	16 (32%)	278 (33.6%)	23 (33.3%)	39 (32.8%)	250 (34.3%)
ECOG performance status score at study entry	​	​	​	​	​	​
0	657 (74.8%)	33 (66%)	624 (75.4%)	52 (75.4%)	85 (71.4%)	549 (75.3%)
≥1	221 (25.2%)	17 (34%)	204 (24.6%)	17 (24.6%)	34 (28.6%)	180 (24.7%)
Baseline PSA (ng/mL) at study entry	​	​	​	​	​	​
<20	480 (54.7%)	20 (40%)	460 (55.6%)	32 (46.4%)	52 (43.7%)	415 (56.9%)
≥20	397 (45.2%)	30 (60%)	367 (44.3%)	37 (53.6%)	67 (56.3%)	313 (42.9%)
Unknown	1 (0.1%)	0 (0%)	1 (0.1%)	0 (0%)	0 (0%)	1 (0.1%)
Alkaline phosphatase (U/L) at study entry	​	​	​	​	​	​
<147	658 (74.9%)	34 (68%)	624 (75.4%)	40 (58%)	74 (62.2%)	584 (80.1%)
≥147	184 (21%)	15 (30%)	169 (20.4%)	25 (36.2%)	40 (33.6%)	144 (19.8%)
Unknown	36 (4.1%)	1 (2%)	35 (4.2%)	4 (5.8%)	5 (4.2%)	1 (0.1%)
Lactate dehydrogenase (U/L) at study entry	​	​	​	​	​	​
<280	584 (66.5%)	36 (72%)	548 (66.2%)	45 (65.2%)	81 (68.1%)	475 (65.2%)
≥280	73 (8.3%)	4 (8%)	69 (8.3%)	10 (14.5%)	14 (11.8%)	58 (8%)
Unknown	221 (25.2%)	10 (20%)	211 (25.5%)	14 (20.3%)	24 (20.2%)	196 (26.9%)
Hemoglobin (g/L) at study entry	​	​	​	​	​	​
≤132	375 (42.7%)	14 (28%)	361 (43.6%)	25 (36.2%)	39 (32.8%)	329 (45.1%)
>132	434 (49.4%)	30 (60%)	404 (48.8%)	36 (52.2%)	66 (55.5%)	345 (47.3%)
Unknown	69 (7.9%)	6 (12%)	63 (7.6%)	8 (11.6%)	14 (11.8%)	55 (7.5%)
Disease extent at study entry	​	​	​	​	​	​
Localized	257 (29.3%)	11 (22%)	246 (29.7%)	19 (27.5%)	30 (25.2%)	227 (31.1%)
Bone only	208 (23.7%)	9 (18%)	199 (24%)	9 (13%)	18 (15.1%)	190 (26.1%)
LN ± bone without visceral	158 (18%)	13 (26%)	145 (17.5%)	16 (23.2%)	29 (24.4%)	129 (17.7%)
Visceral	90 (10.3%)	8 (16%)	82 (9.9%)	11 (15.9%)	19 (16%)	68 (9.3%)
Unknown	165 (18.8%)	9 (18%)	156 (18.8%)	14 (20.3%)	23 (19.3%)	115 (15.8%)
Bone lesions at study entry	​	​	​	​	​	​
≤10	637 (72.6%)	34 (68%)	603 (72.8%)	50 (72.5%)	84 (70.6%)	541 (74.2%)
>10	240 (27.3%)	16 (32%)	224 (27.1%)	19 (27.5%)	35 (29.4%)	187 (25.7%)
Unknown	1 (0.1%)	0 (0%)	1 (0.1%)	0 (0%)	0 (0%)	1 (0.1%)
Gleason score at initial diagnosis	​	​	​	​	​	​
<8	350 (39.9%)	22 (44%)	328 (39.6%)	26 (37.7%)	48 (40.3%)	302 (41.4%)
≥8	430 (49%)	22 (44%)	408 (49.3%)	34 (49.3%)	56 (47.1%)	344 (47.2%)
Unknown	98 (11.2%)	6 (12%)	92 (11.1%)	9 (13%)	15 (12.6%)	83 (11.4%)
Lymph node stage at initial diagnosis	​	​	​	​	​	​
N0	421 (47.9%)	22 (44%)	399 (48.2%)	35 (50.7%)	57 (47.9%)	355 (48.7%)
N1	250 (28.5%)	14 (28%)	236 (28.5%)	18 (26.1%)	32 (26.9%)	201 (27.6%)
Unknown	207 (23.6%)	14 (28%)	193 (23.3%)	16 (23.2%)	30 (25.2%)	173 (23.7%)
Metastasis stage at initial diagnosis	​	​	​	​	​	​
M0	449 (51.1%)	22 (44%)	427 (51.6%)	30 (43.5%)	52 (43.7%)	397 (54.5%)
M1	321 (36.6%)	21 (42%)	300 (36.2%)	32 (46.4%)	53 (44.5%)	238 (32.6%)
Unknown	108 (12.3%)	7 (14%)	101 (12.2%)	7 (10.1%)	14 (11.8%)	94 (12.9%)

Abbreviations: ECOG, Eastern Cooperative Oncology Group; LN, lymph node; PSA, prostate-specific antigen.

### Mutational profiling and biomarker status determination

For targeted panel testing, gene panels were developed at the time of respective studies to investigate HRR pathway alterations in prostate cancer. HRR genes included *ATM*, *BRCA1*, *BRCA2*, *BRIP1*, *CHEK2*, *FANCA*, *HDAC2*, and *PALB2*. Plasma samples from SPARTAN, TITAN, ACIS, and PCR2023 were tested using the targeted HRR gene panel Resolution ctDx HRD (Exact Sciences). Diagnostic tissue samples from LATITUDE were tested using FoundationOne CDx (F1CDx; Foundation Medicine Inc.). The F1CDx panel does not include *HDAC2*. WES was performed on diagnostic tissue and matched normal blood samples from SPARTAN, TITAN, and ACIS.

Targeted panels called both somatic and germline mutations without distinction. For WES, germline and somatic variants were called separately and were combined for this analysis. Pathogenic single-nucleotide variants or deep deletions of germline or somatic origin were used to define HRR gene alteration positivity. No distinction between biallelic or monoallelic alterations was made. A sample was considered *BRCA-alt* if *BRCA*1 or *BRCA*2 gene was altered; similarly, a sample was considered HRR-alt if at least one HRR gene was altered. A patient was considered *BRCA*-*alt* or HRR-alt if at least one of their samples was *BRCA* or HRR altered, respectively. A patient was considered *BRCA-wt* or HRR-wt if *BRCA* or HRR genes were not detected as altered, respectively. Lastly, a patient was considered *BRCA-wt/HRR-alt* if one or more non-*BRCA* HRR genes were detected as altered.

A total of 1,438 samples—including diagnostic tissue and SCRN or EOT blood plasma—were successfully processed. EOT blood samples comprised the largest proportion (39.2%), followed by SCRN blood samples (35.9%) and diagnostic tissue samples (25%).

### WES data processing

WES was successfully performed on 57 SPARTAN, 125 TITAN, and 69 ACIS patient diagnostic FFPE tissue and matched blood samples by Q^2^ Solutions (labs.iqvia.com/). Simultaneous DNA–RNA isolation was achieved from whole blood and FFPE slides using RNA PaxGene blood tubes. The Agilent SureSelect Whole-Exome sequencing kit was used for target capture from input DNA, followed by sequencing on NovaSeq (Illumina). FFPE and blood samples were sequenced paired-end with individual read length of 100 bp and average depth of coverage 100x and 50x, respectively.

Upstream bioinformatics analysis was performed by Q^2^ Solutions’s Tumor–Normal pipeline. In particular, the paired-end reads were aligned to the reference human genome (GRCh38) using Sentieon BWA aligner. Variants found in the matched normal samples were removed from the tumor variant calls to detect somatic mutations using Sentieon TNscope variant caller. Somatic variants were annotated using VarSeq (Golden Helix).

Copy-number variants were called using CNVkit (RRID: SCR_021917). Samples with library complexity <20 million were considered of poor quality and excluded from further analysis. A total of 38 SPARTAN, 97 TITAN, and 64 ACIS matched pairs were retained. Following Seed and colleagues (27), segments with copy ratio log_2_ ≤ −1.2 were considered as deep deletions and log_2_ ≥ 2 as high-level amplifications. Only deep deletions in HRR genes were considered.

Germline variants were called using GATK HaplotypeCaller (RRID: SCR_028440) and annotated using SnpEff (RRID: SCR_005191) from GEMINI (RRID: SCR_014819) on the cloud-based platform by DNAnexus (www.dnanexus.com/; RRID: SCR_011884).

Both germline and somatic variants were first filtered for base call quality (Phred score) and variant call quality. Variants at allelic frequency below 5% were filtered out. Both somatic and germline variants were then filtered to remove population SNPs found in gnomAD (RRID: SCR_014964), NHLBI GO Exome Sequencing Project (RRID: SCR_012761), Exome Aggregation Consortium (RRID: SCR_004068), and 1,000 Genomes (RRID: SCR_006828) at minor allele frequency ≥1%.

Following sequence ontology, all low-impact variants (e.g., synonymous variants or nucleotide changes that do not alter the amino acid sequence) or medium-impact variants (e.g., missense variants or single-nucleotide changes that alter the amino acid sequence) with no evidence of pathogenicity were excluded. High-impact variants (e.g., frameshift variants or insertions or deletions that shift the reading frame, usually resulting in loss of function) from tumor suppressor genes were retained regardless of the evidence of pathogenicity. A medium-impact variant was considered pathogenic if identified as pathogenic or likely pathogenic by ClinVar (www.clinicalgenome.org/data-sharing/clinvar/; RRID: SCR_006169) or present in OncoKB (www.oncokb.org/; RRID: SCR_014782) or Cancer Hotspot (www.cancerhotspots.org/) databases. In addition, a variant was considered pathogenic if predicted damaging by SIFT (RRID: SCR_012813) and possibly damaging or probably damaging by Polyphen (RRID: SCR_013189).

Lastly, germline variants above 85% or between 20% and 65% allelic frequency were retained. Variants present in more than 10% samples per trial were excluded. BRCA2 variants, which were after position 32,972,625, were excluded. Germline variants with low sequencing depth (i.e., <10 for single-base substitutions and <20 for indels) were excluded.

### Clinical outcomes and statistical methodology

Descriptive statistics, including percentages, for baseline characteristics were produced for biomarker groups *BRCA*-alt, *BRCA*-wt/HRR-alt, HRR-alt, and HRR-wt.

The prevalences of overall HRR or *BRCA* alterations per trial and per disease setting were reported. Frequency estimates were accompanied by 95% confidence intervals (CI).

Clinical outcomes assessed were radiographic progression-free survival (rPFS) and overall survival (OS). For SPARTAN, which was conducted in the nmCRPC disease setting, metastasis-free survival was evaluated. For associations between biomarker status and clinical outcomes, Kaplan–Meier curves were displayed. The Cox regression model (multivariate regression with trial as strata) was used for estimating HR, 95% CI, and *P* values. An α level of 0.05 was used for significance. The survival of each biomarker group was compared within trials (the “strata”) by the Cox regression model, and the differences within each stratum were then combined to give an HR that was adjusted for the trial.

## Results

### Baseline demographics and clinical characteristics

In the full cohort of 878 patients, 257 of 878 (29.3%) were nmCRPC, 247 of 878 (28.1%) mCSPC, and 374 of 878 (42.6%) mCRPC. When dichotomized by treatment, 608 of 878 (69.2%) were treated with next-generation ARPI (Apa- or Abi-based regimen plus ADT) and 270 of 878 (30.8%) with ADT alone.

*HDAC2* was not included in the NGS panel used for LATITUDE, in which four of 34 patients had HRR alterations in the remainder of HRR genes. The HRR status could not be concluded for the remaining 30 patients for all eight HRR genes. In the cohort of 848 patients with complete HRR characterization, 50 were *BRCA*-alt, 69 *BRCA*-wt/HRR-alt, and remaining 729 HRR-wt. Baseline characteristics of biomarker groups from the full cohort are described in Table 1. There were no notable differences in baseline demographics and clinical characteristics among biomarker groups.

### Landscape of *BRCA* and other HRR alterations

In the full cohort of 878 patients, 4.3% (95% CI, 2.3–7.7) SPARTAN, 6.6% (95% CI, 3.8–11) TITAN, 2.9% (95% CI, 0.2–17.1) LATITUDE, 6.7% (95% CI, 4–10.8) ACIS, and 5.9% (95% CI, 2.8–11.7) PCR2023 patients were *BRCA*-alt (Supplementary Fig. S1A–S1E). When summarized by disease setting, 4.3% (95% CI, 2.3–7.7) patients with nmCRPC, 6.1% (CI, 3.6–10) patients with mCSPC, and 6.4% (CI, 4.2–9.5) patients with mCRPC were *BRCA*-alt (Fig. 2A–C).

**Figure 2.**
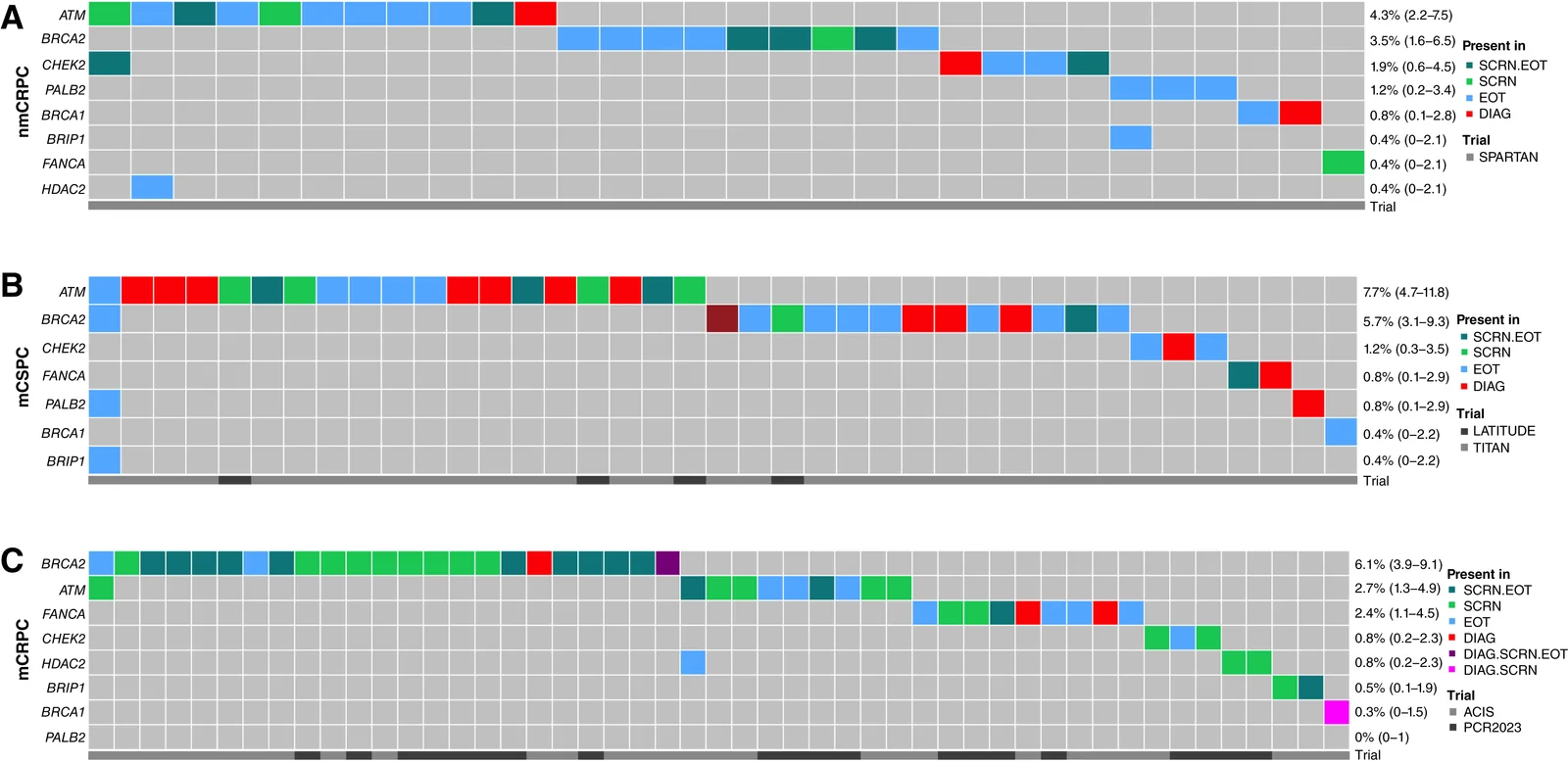
HRR gene alterations across disease settings. **A,** nmCRPC, (**B**) mCSPC, and (**C**) mCRPC. BRCA prevalence is 4.3% (95% CI, 2.3–7.7) in nmCRPC, 6.6% (95% CI, 3.8–11) in mCSPC (**B**), and 6.4% (95% CI, 4.2–9.5) in mCRPC. HRR prevalence is 11.7% (95% CI, 8.14–16.39) in nmCRPC, 16.9% (95% CI, 12.3–22.8) in mCSPC, and 13.1% (95% CI, 9.9–17.1) in mCRPC. Note that *HDAC2* was not included in the NGS panel used for LATITUDE and was therefore excluded from the mCSPC visualization. Frequency calculations were based on the following cohorts: nmCRPC (SPARTAN), mCSPC (TITAN), and mCRPC (ACIS and PCR2023).

Across 844 patients from four trials with complete HRR mutational data, 11.7% (95% CI, 8.14–16.39) SPARTAN, 16.9% (95% CI, 12.3–22.8) TITAN, 12.1% (95% CI, 8.4–17.1) ACIS, and 14.8% (95% CI, 9.5–22.2) PCR2023 patients were HRR-alt (Supplementary Fig. S1A–S1E). When summarized by disease setting, 11.7% (95% CI, 8.14–16.39) patients with nmCRPC, 16.9% (95% CI, 12.3–22.8) patients with mCSPC, and 13.1% (95% CI, 9.9–17.1) patients with mCRPC were HRR-alt (Fig. 2A–C). The prevalence of individual HRR gene alterations across five trials is described in Supplementary Table S1.

Among 71 patients with both diagnostic tissue and blood plasma (SCRN or EOT) samples, plasma and tissue testing demonstrated an overall percent agreement of 97.2% for BRCA alterations and 98.1% for HRR alterations. For BRCA alterations, diagnostic tissue testing showed a positive percent agreement (PPA) of 37.5% and a negative percent agreement (NPA) of 98.9% with blood samples. For HRR alterations, the PPA was 60% and the NPA was 98.5% between diagnostic tissue and blood testing (Fig. 2A–C; Supplementary Fig. S2A and S2B).

### Comparison of clinical outcomes among biomarker groups on next-generation ARPI

In next-generation ARPI–treated patients stratified by *BRCA* status, *BRCA*-alt patients were at increased risk of progression [HR = 1.8 (1.16–2.79); *P* value = 0.009] and mortality [HR = 1.6 (1.02–2.34); *P* value = 0.038] as compared with *BRCA*-wt patients (Fig. 3A and B). When stratified by *BRCA* and HRR status, both *BRCA*-alt and *BRCA*-wt/HRR-alt patients had increased risk of progression compared with HRR-wt patients [*BRCA*-alt: HR = 1.9 (1.2–2.9); *P* value = 0.006 and *BRCA*-wt/HRR-alt: HR = 1.55 (1.01–2.38); *P* value = 0.047]. *BRCA*-alt patients had significantly shorter OS versus HRR-wt patients [*BRCA*-alt: HR = 1.6 (1.03–2.36); *P* value = 0.036 and *BRCA*-wt/HRR-alt: HR = 1.1 (0.68–1.8); *P* value = NS; Fig. 3C and D]. When stratified by HRR status, HRR-alt patients were at increased risk of progression [HR = 1.7 (1.2–2.3); *P* value = 0.001] but not mortality [HR = 1.32 (0.96–1.83); *P* value = ns] compared with HRR-wt patients (Supplementary Fig. S3A and S3B).

**Figure 3.**
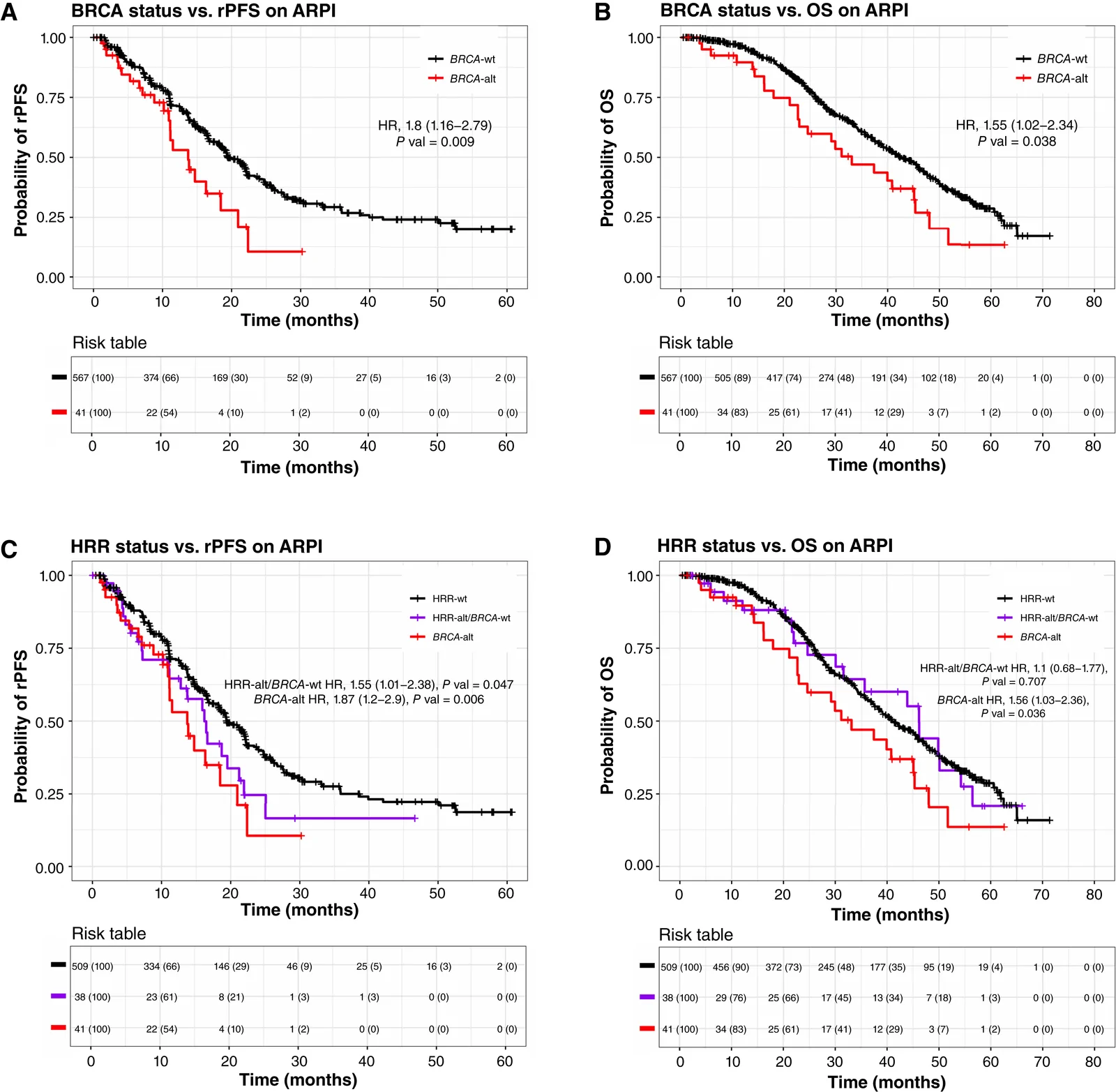
Comparisons of rPFS and OS between biomarker groups in next-generation ARPI–treated patients. **A,** rPFS of *BRCA*-stratified patients. **B,** OS of *BRCA*-stratified patients. **C,** rPFS of *BRCA-* and HRR-stratified patients. **D,** OS of *BRCA-* and HRR-stratified patients. Note that in stratification by *BRCA*, the two arms are *BRCA*-wt and *BRCA*-alt. In stratification by both *BRCA* and HRR, the three arms are HRR-wt, *BRCA*-wt/HRR-alt, and *BRCA*-alt. HRR genes include *BRCA*1, *BRCA*2, *ATM*, *BRIP1*, *CHEK2*, *FANCA*, *HDAC2*, and *PALB2*. For survival analysis, biomarker groups were compared using a stratified Cox regression model, adjusting for trial as strata.

In patients stratified by individual HRR gene alterations, *BRCA*2- and ATM-altered patients were at increased risk of progression [BRCA2: HR = 1.8 (1.14–2.92); *P* value = 0.01 and ATM: HR = 3.1 (1.72–5.59), *P* value = 0.00007] but not mortality [BRCA2: HR = 1.48 (0.95–2.29); *P* value = NS and ATM: HR = 1.51 (0.8–2.84); *P* value = NS] as compared with respective wild-type patients (Supplementary Fig. S3C and S3D). The remaining genes were infrequently altered (nine or fewer patients with alterations in *HDAC2*, *BRCA1*, *BRIP1*, *FANCA*, *CHEK2*, or *PALB2*), precluding reliable statistical analysis for these groups (Supplementary Fig. S3C and S3D).

Lastly, within specific disease settings, when stratified by BRCA or HRR status, patients with BRCA-alt mCRPC were at increased risk of progression [HR = 1.77 (1.02–3.05); *P* value = 0.04] but not mortality [HR = 1.56 (0.92–2.66); *P* value = NS]. Patients with HRR-alt mCRPC were at increased risk of progression [HR = 1.83 (1.24–2.69); *P* value = 0.002] and mortality [HR = 1.56 (1.04–2.34); *P* value = 0.03; Supplementary Fig. S4A–S4D]. Expected trends but lack of significance was observed in nmCRPC [BRCA: rPFS HR = 2.23 (0.68–7.32); *P* value = NS; BRCA: OS HR = 2.28 (0.91–5.69); *P* value = NS and HRR: rPFS HR = 1.64 (0.8–3.34); *P* value = NS; HRR: OS HR = 1.16 (0.59–2.26); *P* value = NS] and mCSPC [BRCA: rPFS HR = 1.68 (0.65–4.34); *P* value = NS; BRCA: OS HR = 1.11 (0.44–2.84); *P* value = NS and HRR: rPFS HR = 1.17 (0.46–3.03); *P* value = NS; HRR: OS HR = 0.83 (0.32–2.1); *P* value = NS; Supplementary Fig. S4A–S4D]. The lack of significant associations in nmCRPC or mCSPC may be attributed to small sample sizes, particularly the limited number of patients with alterations. Specifically, there were 24, 7, and 10 BRCA-altered patients in the mCRPC, nmCRPC, and mCSPC cohorts, respectively, and 49, 17, and 11 HRR-altered patients in the mCRPC, nmCRPC, and mCSPC cohorts, respectively (Supplementary Fig. S4A–S4D).

### Comparison of clinical outcomes among biomarker groups on next-generation ARPI or ADT alone

Association between biomarker status and clinical outcomes was evaluated in all comers to assess the prognostic effect of BRCA or HRR alterations. In next-generation ARPI– or ADT-treated patients stratified by *BRCA* status, *BRCA*-alt patients were at increased risk of progression [HR = 1.6 (1.1–2.4); *P* value = 0.013] and mortality [HR = 1.7 (1.2–2.4); *P* value = 0.005] as compared with *BRCA*-wt patients (Fig. 4A and B). When stratified by *BRCA* and HRR status, both *BRCA*-alt and *BRCA*-wt/HRR-alt patients had increased risk of progression compared with HRR-wt patients [*BRCA*-alt: HR = 1.7 (1.2–2.5); *P* value = 0.007 and *BRCA*-wt/HRR-alt: HR = 1.4 (1.03–2); *P* value = 0.035]. *BRCA*-alt patients had significantly shorter OS than HRR-wt patients [*BRCA*-alt: HR = 1.7 (1.2–2.4); *P* value = 0.004 and *BRCA*-wt/HRR-alt: HR = 1.3 (0.95–1.9); *P* value = NS; Fig. 4C and D]. When stratified by HRR status, HRR-alt patients were at increased risk of progression [HR = 1.5 (1.2–2); *P* value = 0.002] and mortality [HR = 1.5 (1.2–1.9); *P* value = 0.002] as compared with HRR-wt patients (Supplementary Fig. S5A and S5B).

**Figure 4.**
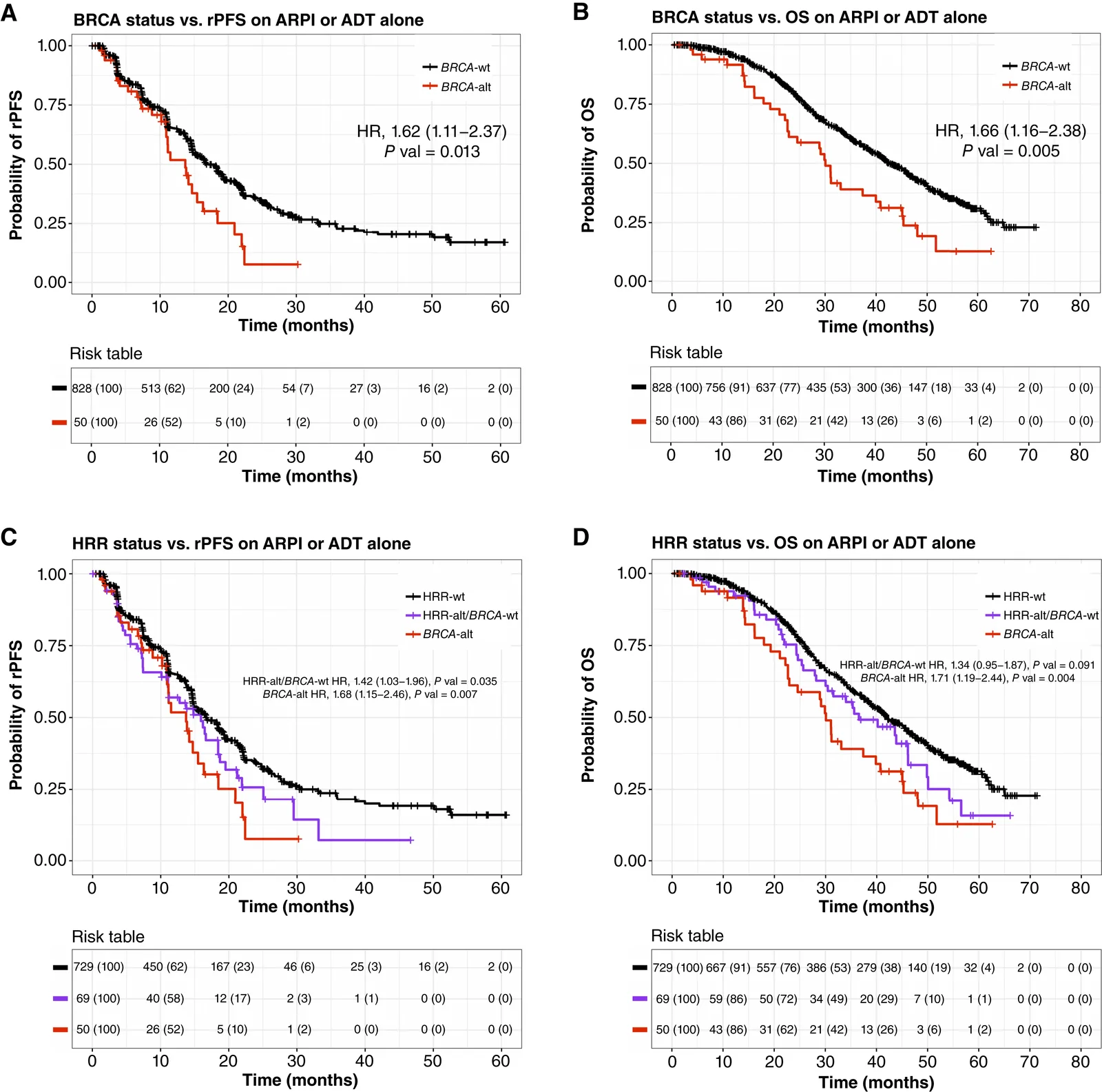
Comparison of rPFS and OS between biomarker groups in next-generation ARPI– or ADT-treated patients. **A,** rPFS of *BRCA*-stratified patients. **B,** OS of *BRCA*-stratified patients. **C,** rPFS of *BRCA-* and HRR-stratified patients. **D,** OS of *BRCA-* and HRR-stratified patients. Note that in stratification by *BRCA*, the two arms are *BRCA*-wt and *BRCA*-alt. In stratification by both *BRCA* and HRR, the three arms are HRR-wt, *BRCA*-wt/HRR-alt, and *BRCA*-alt. HRR genes include *BRCA*1, *BRCA*2, ATM, *BRIP1*, *CHEK2*, *FANCA*, *HDAC2*, and *PALB2*. For survival analysis, biomarker groups were compared using a stratified Cox regression model, adjusting for trial as strata.

In patients stratified by individual HRR gene alterations, *BRCA*2- and ATM-altered patients were at increased risk of progression [BRCA2: HR = 1.72 (1.15–2.56); *P* value = 0.007 and ATM: HR = 2.08 (1.4–3.1); *P* value = 0.0002] and mortality [BRCA2: HR = 1.64 (1.13–2.38); *P* value = 0.009 and ATM: HR = 1.73 (1.15–2.61); *P* value = 0.008] as compared with respective wild-type patients (Supplementary Fig. S5C and S5D). PALB2-altered patients were at increased risk of mortality [HR = 2.84 (1.05–7.66); *P* value = 0.03] but not progression [HR = 2.3 (0.85–6.2); *P* value = NS] as compared with wild-type patients (Supplementary Fig. S5C and S5D). The remaining genes were infrequently altered (12 or fewer patients with alterations in *HDAC2*, *BRCA1*, *BRIP1*, *FANCA*, or *CHEK2*), inhibiting reliable statistical analysis for these groups (Supplementary Fig. S5C and S5D).

Within specific disease settings, when stratified by BRCA or HRR status, patients with BRCA-alt mCRPC were at increased risk of progression [HR = 1.77 (1.02–3.05); *P* value = 0.04] but not mortality [HR = 1.56 (0.92–2.66); *P* value = NS]. Patients with HRR-alt mCRPC were at increased risk of progression [HR = 1.83 (1.24–2.69); *P* value = 0.002] and mortality [HR = 1.56 (1.04–2.34); *P* value = 0.03]. In nmCRPC and mCSPC, similar trends were observed, but statistical significance was generally not reached [nmCRPC: BRCA rPFS HR = 1.34 (0.55–3.3), *P* value = ns; BRCA OS HR = 2.91 (1.47–5.76), *P* value = ns; HRR rPFS HR = 1.36 (0.84–2.2), *P* value = NS; HRR OS HR = 1.96 (1.23–3.13), *P* value = 0.004; mCSPC: BRCA rPFS HR = 1.6 (0.84–3.07), *P* value = NS; BRCA OS HR = 1.24 (0.63–2.44), *P* value = NS; HRR rPFS HR = 1.3 (0.8–2.11), *P* value = ns; HRR OS HR = 1.11 (0.7–1.77), *P* value = NS; Supplementary Fig. S6A–S6D]. The lack of significant associations in nmCRPC and mCSPC may be due to small sample sizes, particularly the limited number of patients with gene alterations. Specifically, there were 49, 30, and 36 HRR-altered patients in the mCRPC, nmCRPC, and mCSPC cohorts, respectively, and 24, 11, and 15 BRCA-altered patients in the mCRPC, nmCRPC, and mCSPC cohorts, respectively (Supplementary Fig. S6A–S6D).

### Comparison of clinical outcomes between treatment groups in *BRCA-alt* or HRR-alt patients

Of 115 HRR-alt patients, 77 were treated with an ARPI-based regimen and 38 with ADT alone. ARPI reduced the risk of progression [HR = 0.42 (0.2–0.89); *P* value = 0.023] and mortality [HR = 0.45 (0.23–0.91); *P* value = 0.025] compared with ADT alone (Fig. 5A). In 50 *BRCA*-alt patients, in whom 41 were treated with an ARPI-based regimen and nine with ADT alone, trends of similar reduction in the risk of progression [HR = 0.44 (0.14–1.42); *P* value = NS] and mortality [HR = 0.64 (0.25–1.66); *P* value = NS] on ARPI versus ADT alone were observed without statistical significance (Fig. 5B). The small sample size, particularly fewer samples in the control arm, may have contributed to lack of statistical significance (Supplementary Fig. S7A and S7B).

**Figure 5.**
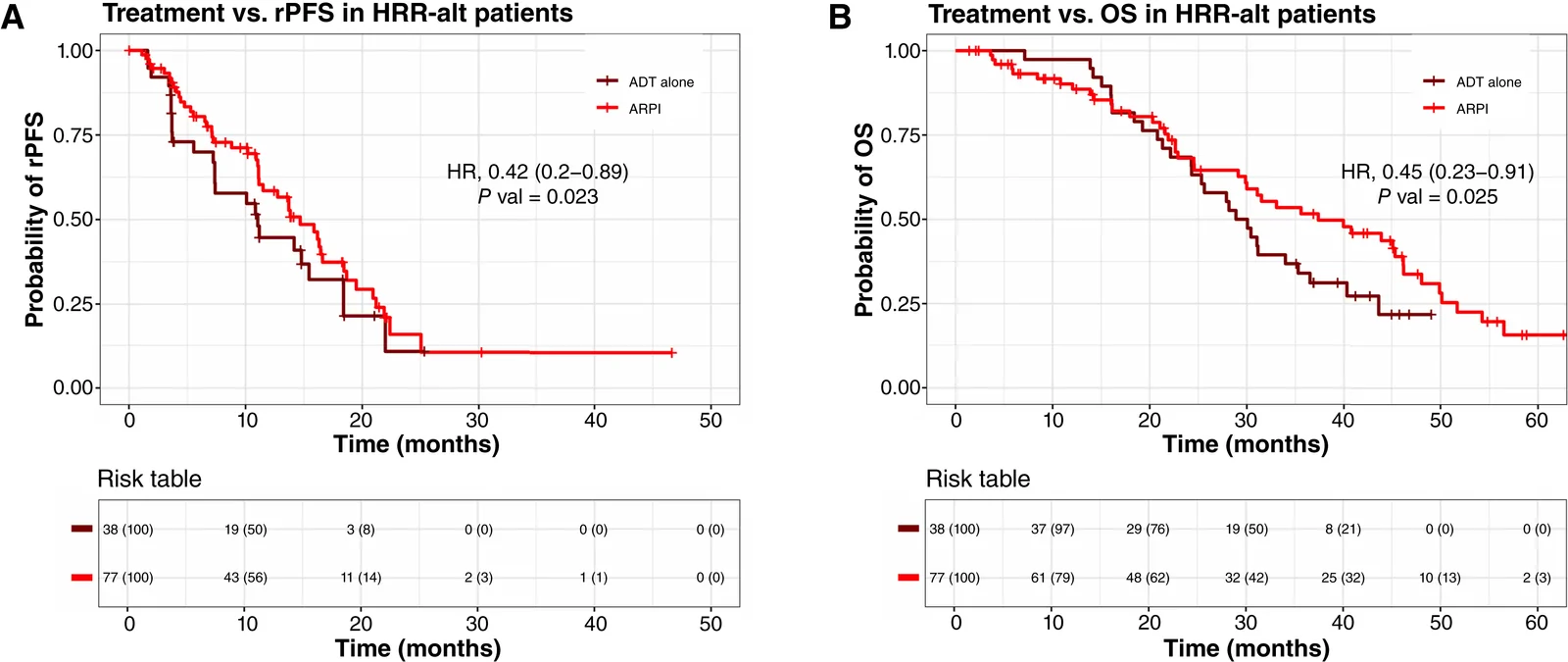
Comparison of rPFS and OS between treatment groups. **A,** rPFS of HRR-alt patients stratified by treatment. **B,** OS of HRR-alt patients stratified by treatment. Note that in stratification by treatment, the two arms are next-generation ARPI or ADT alone. HRR genes include *BRCA*1, *BRCA*2, *ATM*, *BRIP1*, *CHEK2*, *FANCA*, *HDAC2*, and *PALB2*. For survival analysis, biomarker groups were compared using a stratified Cox regression model, adjusting for trial as strata.

The interaction tests between HRR or BRCA status and ARPI treatment effect for rPFS or OS were not statistically significant because of small sample sizes. The results indicate a nonsignificant trend toward reduced benefit of next-generation ARPI treatment for rPFS in BRCA- or HRR-mutated patients [BRCA: HR = 1.1 (0.48–2.79); *P* value = NS and HRR: HR = 1.34 (0.78–2.28); *P* value = NS], and a trend toward greater benefit for OS in these subgroups [BRCA: HR = 0.55 (0.24–1.27); *P* value = NS and HRR: HR = 0.7 (0.41–1.19); *P* value = NS]. These findings should be interpreted with caution because of wide CIs and the lack of statistical significance (Supplementary Table S2).

## Discussion

HRR deficiency is a key molecular mechanism in the tumorigenesis of prostate cancer. Currently, ARPIs are commonly used to treat advanced prostate cancer regardless of HRR alterations. Elucidating the impact of HRR alterations on response to ARPI is important for the clinical management of advanced prostate cancer.

We performed retrospective analysis of pooled data from five randomized trials of next-generation ARPIs (Apa or Abi) in the nmCRPC, mCSPC, and mCRPC disease settings. Patients with diagnostic biopsy tissue or blood plasma collected at SCRN or EOT were included in the analysis. Several efforts analyzing primary prostate tumor and metastatic tissue have suggested that HRR alterations occur early in tumorigenesis (4, 5, 28). Similarly, we observed modest concordance in BRCA or HRR alterations between diagnostic biopsy tissue and blood plasma (SCRN or EOT) samples. Our testing procedure did not distinguish between biallelic versus monoallelic HRR variants. Systematic studies comparing the utility of *BRCA* as a prognostic marker in mCRPC have highlighted its utility independent of zygosity (29). These findings reinforce the validity of our study design.

Moreover, targeted sequencing panels for ctDNA and tissue testing do not distinguish between germline and somatic variants. For WES, somatic and germline variants were called using separate pipelines but combined early in the workflow. Notably, exploratory studies indicated no significant difference in clinical outcomes in patients with somatic or germline BRCA variants (30).

Our study indicates that *BRCA*-alt patients are at higher risk of progression and mortality than *BRCA*-wt patients on AR-directed treatment. Although HRR-alt patients do benefit from the addition of ARPIs to ADT, they nevertheless exhibit worse clinical outcomes than patients without HRR alterations on AR-targeted therapy. Similarly, patients with HRR gene alterations other than BRCA also had shorter PFS than HRR-wt patients. However, only BRCA gene–altered patients had significantly shorter OS compared with HRR-wt patients. In another recent study, patients with HRR alterations other than BRCA had shorter OS than HRR-wt on first-line mCRPC treatment with ARPI or taxane (31).

In particular, among 115 HRR-alt patients, the addition of next-generation ARPI to ADT significantly prolonged PFS and OS relative to treatment with ADT alone. Similar analysis on *BRCA*-alt patients showed trends of reduced risk of progression and mortality in ARPI plus ADT versus ADT alone but they did not reach significance likely because of very few *BRCA*-alt patients. These results highlight the relevance of treating the AR axis in HRR-alt patients with intensification of next-generation ARPI but indicate that the patients require additional therapeutic approaches that address the impact of HRR alterations for better outcomes.

Our study has a few limitations. First, samples collected at different timepoints were pooled to determine the biomarker status per patient; in particular, EOT plasma samples were used to define alteration status in some cases. However, several efforts analyzing primary prostate tumor and metastatic tissue have suggested that HRR alterations occur early in tumorigenesis; thus, those patients positive for BRCA or HRR at EOT are unlikely to have these mutations acquired on study treatment (4, 5, 28). While acknowledging this limitation, the main objective of our study was to assess the impact of *BRCA* or HRR alterations on ARPI treatment outcome in a retrospective manner. Thus, the biomarker status was derived using all the available evidence of biomarker status for a given patient.

Second, HRR alteration frequencies reported in our analysis may seem slightly lower in comparison with the frequencies observed in prostate cancer studies [e.g., BRCA1/2 alteration frequency in Stand Up To Cancer at 11.7% (CI, 8.9–15.2) vs. our mCRPC cohort at 6.4% (CI, 4.2–9.5) vs. our nmCRPC cohort at 4.3% (CI, 2.3–7.7); refs. 5, 6]. These differences can be partially explained by different disease settings as patients who respond poorly to SOC therapies are more likely to develop advanced (metastatic) disease. Additionally, tissue sample testing may offer higher sensitivity for detecting copy-number alterations in contrast to plasma testing (32), whereas ctDNA testing is less invasive and more sensitive with increased disease burden. In that regard, our methodologic approach is consistent with current real-world clinical practice. Our SCRN plasma samples might contribute to lower prevalences likely because of low disease burden at the trial start (Supplementary Fig. S2A and S2B). Clinical trials often account for this limitation by testing multiple samples per patient (14, 16). Moreover, tumor fraction (TF) estimation can be used to adjust for confounding factors related to low disease burden or limited tumor shedding. However, TF measurement is typically performed using whole-genome sequencing, which was not employed in our study and therefore represents a limitation of our analysis.

Furthermore, our HRR gene list might not be comprehensive and includes *ATM*, a debated gene in relation to HRR-related outcomes to PARPi. *ATM* is known to play a key role in DNA repair and cell-cycle response following DNA damage (33), and *ATM* has been included in PARPi trial gene lists for patient selection (15, 16, 33). Our analyses demonstrated significant association between *ATM* alterations and shorter rPFS and OS in subjects treated with ARPI or ADT.

Finally, although we find our study design powerful for covering the full spectrum of advanced prostate cancer, combined disease setting studies are not suitable to measure the median time to events. The median time to progression on specific ARPIs in individual disease settings is well captured in prospective trials (14–16). Our study complements these trials by systematically comparing the outcomes in biomarker groups through a pooled design powered for statistical analysis. Moreover, to systematically combine patients of different disease settings and treatment regimens, differences in trials were controlled.

Our study demonstrates the impact of BRCA (or HRR) on AR-directed treatment outcomes. We effectively utilized archival and blood plasma samples to determine biomarker status. We report that although patients with HRR alterations benefit from the addition of next-generation ARPI to ADT, having such a gene alteration portends more rapid progression on AR-targeted therapy, in particular for patients with BRCA alterations. By combining patients from different disease settings in about equal proportion, our study emphasizes that the power of BRCA as a biomarker goes beyond a particular disease setting in prostate cancer. Our study underlines the unmet need for new and combination approaches for *BRCA*-alt patients on AR-directed treatments in the prostate cancer disease spectrum.

## Supplementary Material

Supplementary Figure S1 Mutational landscape of HRR alterations per trial. (A) SPARTAN, (B) TITAN, (C) LATITUDE, (D) ACIS, (E) PCR2023

Supplementary Figure S2 Comparison of variant detection rates across sample types.

Supplementary Figure S3 Comparison of rPFS or OS between HRR gene–altered and wild-type groups (all HRR alterations combined or by individual HRR gene) in patients receiving ARPI.

Supplementary Figure S4 Comparison of rPFS or OS between BRCA or HRR altered and wildtype groups across disease settings on ARPI.

Supplementary Figure S5 Comparison of rPFS or OS between HRR gene–altered and wild-type groups (all HRR alterations combined or by individual HRR gene) in patients receiving ARPI or ADT alone.

Supplementary Figure S6 Comparison of rPFS or OS between BRCA or HRR altered and wildtype groups across disease settings on ARPI or ADT alone.

Supplementary Figure S7 Comparison of rPFS or OS between treatment groups.

Supplementary Table S1 The prevalence of HRR gene alterations. The frequency of HRR gene mutations is displayed with 95% CI. Note that HDAC2 was not included in the panel used for testing LATITUDE samples.

Supplementary Table S2 The results of interaction tests are displayed.

## Data Availability

The data-sharing policy of Johnson & Johnson is available at https://innovativemedicine.jnj.com/our-innovation/clinical-trials/transparency. As noted on this site, requests for access to the study data can be submitted through Yale Open Data Access Project site at http://yoda.yale.edu.
