# Supplementary material for: Impact of BRCA Alterations on Androgen Receptor Pathway Inhibition Treatment Outcomes in Advanced Prostate Cancer
Source: Cancer Res Commun. 2026 Oct 12;6(10):2371–81. doi: 10.1158/2767-9764.CRC-26-0477 (PMC13656304; doi:10.1158/2767-9764.CRC-26-0477)
Supplement: Supplementary file 1 [file crc-26-0477_supplementary_figure_s1_suppsf1.docx]

**Supplementary Figure S1. Mutational landscape of HRR alterations per trial. (A) SPARTAN, (B) TITAN, (C) LATITUDE, (D) ACIS, (E) PCR2023**

DIAG, diagnostic tissue sample; EOT, end of treatment plasma sample; HRR, homologous recombination repair; SCRN, screening plasma sample.
