# Supplementary material for: Impact of BRCA Alterations on Androgen Receptor Pathway Inhibition Treatment Outcomes in Advanced Prostate Cancer
Source: Cancer Res Commun. 2026 Oct 12;6(10):2371–81. doi: 10.1158/2767-9764.CRC-26-0477 (PMC13656304; doi:10.1158/2767-9764.CRC-26-0477)
Supplement: Supplementary file 2 [file crc-26-0477_supplementary_figure_s2_suppsf2.docx]

**Supplementary Figure S2. Comparison of variant detection rates across sample types.**

(A) Only subjects (N=363) with both SCRN and EOT plasma samples are included. Across 2904 tests (i.e., 363 subjects x 8 genes), overall percent agreement (OPA) of 98.9% between SCRN and EOT. SCRN testing showed positive percent agreement (PPA) of 54% and negative percent agreement (NPA) of 99.7% with EOT. Across 726 tests (i.e., 363 subjects x 2 BRCA genes), OPA of 98.6% between SCRN and EOT. SCRN testing showed PPA of 68.2% and NPA of 99.6% with EOT. (B) Only subjects (N=71) with both DIAG and SCRN or EOT plasma samples are explored. Across 568 tests (i.e., 71 subjects x 8 genes), OPA of 98.1% between DIAG and SCRN/EOT. SCRN/EOT testing showed PPA of 37.5% and NPA of 98.9% with DIAG. Across 142 tests (i.e., 71 subjects x 2 BRCA genes), OPA of 97.2% between DIAG and SCRN/EOT. SCRN/EOT testing showed PPA of 60% and NPA of 98.5% with DIAG.

DIAG, diagnostic tissue sample; EOT, end of treatment plasma sample; HRR, homologous recombination repair; SCRN, screening plasma sample.
