# Supplementary material for: Impact of BRCA Alterations on Androgen Receptor Pathway Inhibition Treatment Outcomes in Advanced Prostate Cancer
Source: Cancer Res Commun. 2026 Oct 12;6(10):2371–81. doi: 10.1158/2767-9764.CRC-26-0477 (PMC13656304; doi:10.1158/2767-9764.CRC-26-0477)
Supplement: Supplementary file 3 [file crc-26-0477_supplementary_figure_s3_suppsf3.docx]

**Supplementary Figure S3. Comparison of rPFS or OS between HRR gene–altered and wild-type groups (all HRR alterations combined or by individual HRR gene) in patients receiving ARPI.**

(A) rPFS of HRR stratified patients on next-generation ARPI. (B) OS of HRR stratified patients on next-generation ARPI. (C) rPFS of HRR gene stratified patients on next-generation ARPI. (D) OS of HRR gene stratified patients on next-generation ARPI. Note that in stratification by HRR, the two arms are HRR wildtype (HRR-wt) and HRR altered (HRR-alt). HRR genes include BRCA1, BRCA2, ATM, BRIP1, CHEK2, FANCA, HDAC2, and PALB2. For survival analysis, biomarker groups were compared using a stratified cox regression model, adjusting for trial as strata.

ADT, androgen deprivation therapy; alt, altered; ARPI, androgen receptor pathway inhibitors; CI, confidence interval; HR, hazard ratio; HRR, homologous recombination repair; rPFS, radiographic progression free survival; OS, overall survival; rPFS, radiographic progression free survival; wt, wild type.
