# Supplementary material for: Impact of BRCA Alterations on Androgen Receptor Pathway Inhibition Treatment Outcomes in Advanced Prostate Cancer
Source: Cancer Res Commun. 2026 Oct 12;6(10):2371–81. doi: 10.1158/2767-9764.CRC-26-0477 (PMC13656304; doi:10.1158/2767-9764.CRC-26-0477)
Supplement: Supplementary file 5 [file crc-26-0477_supplementary_figure_s5_suppsf5.docx]

**Supplementary Figure S5. Comparison of rPFS or OS between HRR gene–altered and wild-type groups (all HRR alterations combined or by individual HRR gene) in patients receiving ARPI or ADT alone.**

(A) rPFS of HRR stratified patients on next-generation ARPI or ADT alone. (B) OS of HRR stratified patients on next-generation ARPI or ADT alone. (C) rPFS of HRR gene stratified patients on next-generation ARPI or ADT alone. (D) OS of HRR gene stratified patients on next-generation ARPI or ADT alone. HRR genes include BRCA1, BRCA2, ATM, BRIP1, CHEK2, FANCA, HDAC2, and PALB2. For survival analysis, biomarker groups were compared using a stratified cox regression model, adjusting for trial as strata.

CI, confidence interval; HR, hazard ratio; HRR, homologous recombination repair; OS, overall survival; rPFS, radiographic progression free survival. For survival analysis, biomarker groups were compared using a stratified cox regression model, adjusting for trial as strata.
