# Supplementary material for: Impact of BRCA Alterations on Androgen Receptor Pathway Inhibition Treatment Outcomes in Advanced Prostate Cancer
Source: Cancer Res Commun. 2026 Oct 12;6(10):2371–81. doi: 10.1158/2767-9764.CRC-26-0477 (PMC13656304; doi:10.1158/2767-9764.CRC-26-0477)
Supplement: Supplementary file 6 [file crc-26-0477_supplementary_figure_s6_suppsf6.docx]

**Supplementary Figure S6. Comparison of rPFS or OS between BRCA or HRR altered and wildtype groups across disease settings on ARPI or ADT alone.**

(A) rPFS of BRCA stratified patients on ARPI or ADT alone across disease settings. (B) OS of BRCA stratified patients on ARPI or ADT alone across disease settings. (C) rPFS of HRR stratified patients on ARPI or ADT alone across disease settings. (D) OS of HRR stratified patients on ARPI or ADT alone across disease settings.

CI, confidence interval; HR, hazard ratio; HRR, homologous recombination repair; OS, overall survival; rPFS, radiographic progression free survival. For survival analysis, biomarker groups were compared using a stratified cox regression model, adjusting for trial as strata.
