# Supplementary material for: Impact of BRCA Alterations on Androgen Receptor Pathway Inhibition Treatment Outcomes in Advanced Prostate Cancer
Source: Cancer Res Commun. 2026 Oct 12;6(10):2371–81. doi: 10.1158/2767-9764.CRC-26-0477 (PMC13656304; doi:10.1158/2767-9764.CRC-26-0477)
Supplement: Supplementary file 7 [file crc-26-0477_supplementary_figure_s7_suppsf7.docx]

**Supplementary Figure S7. Comparison of rPFS or OS between treatment groups.**

(A) rPFS of BRCA altered patients stratified by treatment. (B) OS of BRCA altered patients stratified by treatment.

CI, confidence interval; HR, hazard ratio; HRR, homologous recombination repair; OS, overall survival; rPFS, radiographic progression free survival. For survival analysis, biomarker groups were compared using a stratified cox regression model, adjusting for trial as strata.
