# Supplementary material for: Impact of BRCA Alterations on Androgen Receptor Pathway Inhibition Treatment Outcomes in Advanced Prostate Cancer
Source: Cancer Res Commun. 2026 Oct 12;6(10):2371–81. doi: 10.1158/2767-9764.CRC-26-0477 (PMC13656304; doi:10.1158/2767-9764.CRC-26-0477)
Supplement: Supplementary file 8 [file crc-26-0477_supplementary_table_s1_suppst1.docx]

**Supplementary Table S1. The prevalence of HRR gene alterations. The frequency of HRR gene mutations is displayed with 95% CI. Note that HDAC2 was not included in the panel used for testing LATITUDE samples.**

| **TRIAL** | **SPARTAN** | **TITAN** | **LATITUDE** | **ACIS** | **PCR2023** |
| --- | --- | --- | --- | --- | --- |
| **DISEASE** | nmCRPC | mCSPC | mCSPC | mCRPC | mCRPC |
| **N** | 257 | 213 | 34 | 239 | 135 |
| **ATM** | 4.3% [2.3–7.7%] | 7.5% [4.5–12.1%] | 8.8% [2.3–24.8%] | 2.5% [1–5.6%] | 3.0% [1–7.9%] |
| **BRCA1** | 0.8% [0.1–3.1%] | 0.5% [0–3.0%] | 0% [0–12.6%] | 0.4% [0–2.7%] | 0% [0–3.4%] |
| **BRCA2** | 3.5% [1.7–6.8%] | 6.1% [3.4–10.4%] | 2.9% [0.2–17.1%] | 6.3% [3.7–10.3%] | 5.9% [2.8–11.7%] |
| **BRIP1** | 0.4% [0–2.5%] | 0.5% [0–3.0%] | 0% [0–12.6%] | 0.8% [0.1–3.3%] | 0% [0–3.4%] |
| **CHEK2** | 1.9% [0.7–4.7%] | 1.4% [0.4–4.4%] | 0% [0–12.6%] | 0.4% [0–2.7%] | 1.5% [0.3–5.8%] |
| **FANCA** | 0.4% [0–2.5%] | 0.9% [0.2–3.7%] | 0% [0–12.6%] | 2.1% [0.8–5.1%] | 3.0% [1–7.9%] |
| **HDAC2** | 0.4% [0–2.5%] | 1.4% [0.4–4.4%] | NA | 0.4% [0–2.7%] | 1.5% [0.3–5.8%] |
| **PALB2** | 1.2% [0.3–3.7%] | 0.9% [0.2–3.7%] | 0% [0–12.6%] | 0% [0–2.0%] | 0% [0–3.4%] |

CI, confidence interval
