# Supplementary material for: Impact of BRCA Alterations on Androgen Receptor Pathway Inhibition Treatment Outcomes in Advanced Prostate Cancer
Source: Cancer Res Commun. 2026 Oct 12;6(10):2371–81. doi: 10.1158/2767-9764.CRC-26-0477 (PMC13656304; doi:10.1158/2767-9764.CRC-26-0477)
Supplement: Supplementary file 9 [file crc-26-0477_supplementary_table_s2_suppst2.docx]

**Supplementary Table S2 The results of interaction tests are displayed.**

| **Interaction tests** |  |  |  |  |
| --- | --- | --- | --- | --- |
| **BRCA, rPFS, interaction test** | | | | |
|  | **exp(coef)** | **lower .95** | **upper .95** | **Pr(>\|z\|)** |
| BRCAMUTATED | 1.5967478 | 0.7455982 | 3.419541 | 0.2284317 |
| ARMAPA.ABI | 0.4176134 | 0.3201119 | 0.5448125 | 0 |
| BRCAMUTATED:ARMAPA.ABI | 1.1630155 | 0.4843468 | 2.7926376 | 0.735442 |
|  |  |  |  |  |
| **BRCA, OS, interaction test** | | | | |
|  | **exp(coef)** | **lower .95** | **upper .95** | **Pr(>\|z\|)** |
| BRCAMUTATED | 2.7023053 | 1.3179823 | 5.540632 | 0.0066546 |
| ARMAPA.ABI | 0.942682 | 0.7315172 | 1.214803 | 0.6482652 |
| BRCAMUTATED:ARMAPA.ABI | 0.5540153 | 0.2425404 | 1.265492 | 0.1611335 |
|  |  |  |  |  |
| **HRR, rPFS, interaction test** | | | | |
|  | **exp(coef)** | **lower .95** | **upper .95** | **Pr(>\|z\|)** |
| HRRMUTATED | 1.2677377 | 0.8246293 | 1.948947 | 0.2796141 |
| ARMAPA.ABI | 0.4130209 | 0.3123043 | 0.5462182 | 0 |
| HRRMUTATED:ARMAPA.ABI | 1.3364483 | 0.7823588 | 2.2829602 | 0.2884355 |
|  |  |  |  |  |
| **HRR, OS, interaction test** | | | | |
|  | **exp(coef)** | **lower .95** | **upper .95** | **Pr(>\|z\|)** |
| HRRMUTATED | 1.8836955 | 1.2333225 | 2.877032 | 0.0033846 |
| ARMAPA.ABI | 0.9884969 | 0.7582422 | 1.288673 | 0.9318542 |
| HRRMUTATED:ARMAPA.ABI | 0.6992537 | 0.410518 | 1.19107 | 0.1880056 |
